# Unexpected subcellular distribution of a specific isoform of the Coxsackie and adenovirus receptor, CAR-SIV, in human pancreatic beta cells

**DOI:** 10.1007/s00125-018-4704-1

**Published:** 2018-08-03

**Authors:** Eseoghene Ifie, Mark A. Russell, Shalinee Dhayal, Pia Leete, Guido Sebastiani, Laura Nigi, Francesco Dotta, Varpu Marjomäki, Decio L. Eizirik, Noel G. Morgan, Sarah J. Richardson

**Affiliations:** 1 Islet Biology Exeter (IBEx), Institute of Biomedical and Clinical Sciences, University of Exeter Medical School, RILD Building (Level 4), Barrack Road, Exeter, EX2 5DW UK; 2 Department of Medicine, Surgery and Neurosciences, University of Siena and Fondazione Umberto Di Mario ONLUS—Toscana Life Sciences, Siena, Italy; 3 Department of Biological and Environmental Science/Nanoscience Center, University of Jyväskylä, Jyväskylä, Finland; 4 Université Libre de Bruxelles (ULB) Center for Diabetes Research and Welbio, Medical Faculty, Université Libre de Bruxelles, Brussels, Belgium

**Keywords:** Beta cells, Coxsackie and adenovirus receptor, Coxsackievirus B, Enterovirus, Insulin granule, Pancreas, Protein interacting with C-kinase 1 (PICK1)

## Abstract

**Aims/hypothesis:**

The Coxsackie and adenovirus receptor (CAR) is a transmembrane cell-adhesion protein that serves as an entry receptor for enteroviruses and may be essential for their ability to infect cells. Since enteroviral infection of beta cells has been implicated as a factor that could contribute to the development of type 1 diabetes, it is often assumed that CAR is displayed on the surface of human beta cells. However, CAR exists as multiple isoforms and it is not known whether all isoforms subserve similar physiological functions. In the present study, we have determined the profile of CAR isoforms present in human beta cells and monitored the subcellular localisation of the principal isoform within the cells.

**Methods:**

Formalin-fixed, paraffin-embedded pancreatic sections from non-diabetic individuals and those with type 1 diabetes were studied. Immunohistochemistry, confocal immunofluorescence, electron microscopy and western blotting with isoform-specific antisera were employed to examine the expression and cellular localisation of the five known CAR isoforms. Isoform-specific qRT-PCR and RNA sequencing (RNAseq) were performed on RNA extracted from isolated human islets.

**Results:**

An isoform of CAR with a terminal SIV motif and a unique PDZ-binding domain was expressed at high levels in human beta cells at the protein level. A second isoform, CAR-TVV, was also present. Both forms were readily detected by qRT-PCR and RNAseq analysis in isolated human islets. Immunocytochemical studies indicated that CAR-SIV was the principal isoform in islets and was localised mainly within the cytoplasm of beta cells, rather than at the plasma membrane. Within the cells it displayed a punctate pattern of immunolabelling, consistent with its retention within a specific membrane-bound compartment. Co-immunofluorescence analysis revealed significant co-localisation of CAR-SIV with zinc transporter protein 8 (ZnT8), prohormone convertase 1/3 (PC1/3) and insulin, but not proinsulin. This suggests that CAR-SIV may be resident mainly in the membranes of insulin secretory granules. Immunogold labelling and electron microscopic analysis confirmed that CAR-SIV was localised to dense-core (insulin) secretory granules in human islets, whereas no immunolabelling of the protein was detected on the secretory granules of adjacent exocrine cells. Importantly, CAR-SIV was also found to co-localise with protein interacting with C-kinase 1 (PICK1), a protein recently demonstrated to play a role in insulin granule maturation and trafficking.

**Conclusions/interpretation:**

The SIV isoform of CAR is abundant in human beta cells and is localised mainly to insulin secretory granules, implying that it may be involved in granule trafficking and maturation. We propose that this subcellular localisation of CAR-SIV contributes to the unique sensitivity of human beta cells to enteroviral infection.

**Electronic supplementary material:**

The online version of this article (10.1007/s00125-018-4704-1) contains peer-reviewed but unedited supplementary material, which is available to authorised users.

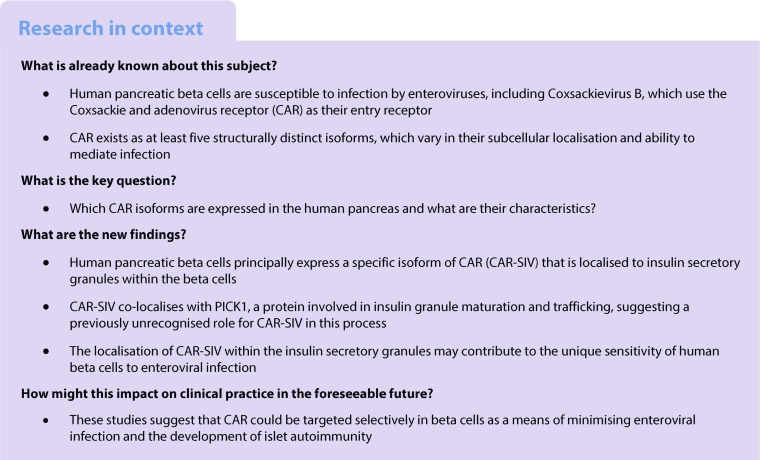



## Introduction

Epidemiological and pathological studies have linked enteroviral infections with the development of type 1 diabetes mellitus [1], but the mechanisms by which this might promote islet autoimmunity remain uncertain. One possibility is that islet cells are particularly sensitive to infection by enteroviruses and, in accord with this, enterovirus serotypes that are most often associated with type 1 diabetes (e.g. Coxsackievirus B [CVB]) have a clear tropism for human beta cells [2–4]. Additional support comes from evidence that enterovirus proteins are present in the beta cells of individuals with type 1 diabetes [5, 6] and that specific viral response pathways are activated in such cells [7, 8].

In order for enteroviruses to infect cells, they must bind to specific cell surface proteins that serve as vehicles to mediate their entry. Such binding is thought to alter the conformation of the viral capsid to facilitate the entry of viral RNA into the target cell. An endogenous cellular protein known as the Coxsackie and adenovirus receptor (CAR) is one such viral receptor. CAR is a transmembrane protein involved in homotypic cell adhesion and tight junctional integrity [9] but also serves as a primary cellular attachment protein for CVBs and adenoviruses [10], presumably in a subversion of its normal physiological role.

**Fig. 1:**
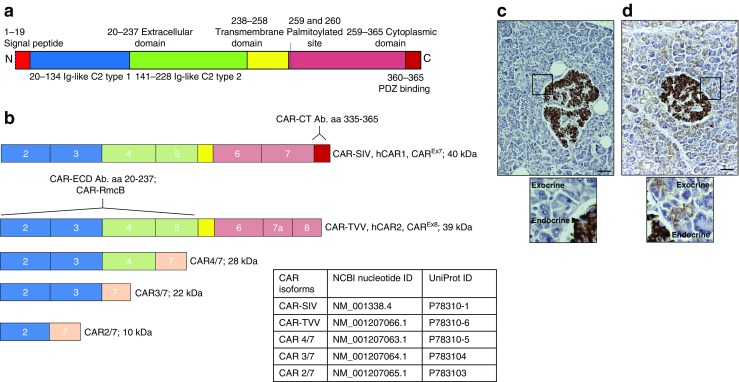
A description of the CAR isoforms and the selective expression of CAR-SIV in human islets. (**a**) CAR protein structure. The signal peptide (red) is cleaved to yield a mature protein with an ECD comprising two immunoglobulin (Ig)-like domains, type 1 (blue) and type 2 (green). The transmembrane domain (yellow) bridges the extracellular and cytoplasmic regions (pink), which terminates with a PDZ-binding domain (red). (**b**) *CXADR* exon maps of the five differentially spliced isoforms. The type 1 Ig domain is encoded by exons 2 and 3, while type 2 Ig-like domain is encoded by exons 4 and 5. Isoforms 1 and 2 contain a transmembrane domain and are named CAR-SIV (or hCAR1, CAR^Ex7^) and CAR-TVV (or hCAR2, CAR^Ex8^), respectively (denoted by the three terminal amino acids on their C-termini). The soluble isoforms 3, 4 and 5 are named CAR4/7, CAR3/7 and CAR2/7, respectively, reflecting exon inclusion or exclusion and lack of the transmembrane domain. The binding regions of the different CAR antibodies are also shown. The CAR-CT antiserum recognises amino acids 335–365 located at the C-terminus of the CAR-SIV isoform but does not recognise the other CAR isoforms. Both the CAR-ECD and CAR-RmcB antisera recognise epitopes within the ECD, and are predicted to recognise the majority of the longer CAR isoforms (CAR-SIV, CAR-TVV, CAR4/7 and potentially CAR3/7). (**c**, **d**) Representative immunocytochemical analysis of the expression of different CAR isoforms in normal control pancreas tissue stained with (**c**) CAR-CT and (**d**) CAR-ECD antisera. Scale bars, 20 μm. The insets below represent higher magnification images (of the areas outlined by the black boxes) demonstrating the differential staining profile of the CAR-CT antisera in the endocrine (+++) and exocrine pancreas (−), and the CAR-ECD antisera in the endocrine (+++) and exocrine pancreas (+); +++, strong; +, weak; −, negative. These results are representative of findings in pancreas from eight non-diabetic individuals; aa, amino acids, Ab., antibody

Full-length CAR is encoded by the *CXADR* gene, which comprises eight exons and yields a protein with an extracellular domain (ECD) linked by a single transmembrane region to a cytoplasmic tail. Differential splicing yields at least five different isoforms (Fig. 1a,b), but only two of these contain the transmembrane domain and are likely to be retained within cells. Structural studies have suggested that enteroviruses bind to the D1 domain in the extracellular region of the protein [11, 12] and, accordingly, four of the isoforms (designated CAR-SIV, CAR-TVV, CAR4/7 and CAR3/7) have been shown to bind enterovirus. However, only CAR-SIV and CAR-TVV retain the transmembrane domain and are thus able to mediate a productive infection in cells. The two soluble isoforms are released from cells and may protect from infection by sequestering active virus in the extracellular fluid [11].

Interestingly, the two isoforms bearing a transmembrane domain vary only in the sequence of the final 26 (CAR-SIV) or 13 (CAR-TVV) amino acids, but are differentially localised within cells and may fulfil different functions [13]. The CAR-SIV isoform (also known as CAR^Ex7^ [13]) is encoded by the first seven exons of the human *CXADR* gene and is expressed preferentially on the basolateral surface of polarised cells. In contrast, the C-terminal region of the CAR-TVV isoform (also known as CAR^Ex8^) is produced from a cryptic splice site within the seventh exon linked to exon 8, and is expressed apically [13]. The C-terminal region encodes a consensus PDZ-binding domain whose amino acid sequence varies between the CAR-SIV and the CAR-TVV forms, which probably accounts for the differential localisation of the two variants within polarised cells [13–16]. In support of this, both the SIV and TVV forms of CAR can interact with proteins such as membrane-associated guanylate kinase, WW and PDZ domain-containing protein 1b (MAGI-Ib), postsynaptic density protein 95 (PSD-95) [13, 15, 16] and zonula occludens-1 (ZO-1) [14], but only the CAR-SIV isoform interacts with protein interacting with C-kinase 1 (PICK1) [13]. Strikingly, PICK1 is abundantly expressed in beta cells and has recently been shown to control insulin granule trafficking. Indeed, loss of PICK1 results in impaired glucose tolerance and reduced serum insulin concentrations in experimental animals [17, 18]. Thus, an interaction between PICK1 and the SIV isoform of CAR in beta cells could be of functional importance.

To date, few studies have examined the expression and distribution of CAR isoforms in human beta cells [3, 19]. Ylipaasto et al [3] confirmed a role for CAR by demonstrating that pre-treatment of isolated human islets with a blocking antibody (clone RmcB) protected the cells from CVB infection [3]. More recently, CAR expression was confirmed in human pancreas, albeit using an antiserum that does not discriminate between the various isoforms [20]. We have thus characterised and mapped the expression and distribution of CAR isoforms in human pancreas.

## Methods

### Tissue and cell lines

Formalin-fixed, paraffin-embedded (FFPE) pancreatic sections from the Exeter Archival Diabetes Biobank (http://foulis.vub.ac.be/) and from the Network for Pancreatic Organ donors with Diabetes (nPOD; Gainesville, FL, USA) were studied. Samples were from 21 non-diabetic individuals (age range 4–47 years) and ten individuals with type 1 diabetes (age range 6–47 years; see electronic supplementary material [ESM] Table 1). Isolated human islets were obtained from the Oxford Centre for Islet Transplantation (Oxford, UK) or purchased from Lonza (Basel, Switzerland). On arrival, islets were cultured for 24 h at 37°C and then fixed and processed for immunostaining by standard immunofluorescence (FFPE) techniques, or stored at −80°C for RNA extraction. Ethics approval (West of Scotland Research Committee 4 [WoSREC4]; 15/WS/0258) was gained for all the samples studied. The pancreatic tissue samples from the Exeter Archival Diabetes Biobank and nPOD were selected based on age of demise, and in the case of type 1 diabetes, duration of disease; tissue quality; sample availability (which is restricted within these biobanks) and appropriateness of tissue for immunohistochemistry/immunofluorescence approaches. Samples with extensive autolysis or evidence of other unrelated pancreatic diseases were excluded. Blinding of donor type and immunofluorescence stain was performed for the co-localisation studies. The human beta cell lines EndoC-βH1 and 1.1B4 were cultured as described in ESM Methods.

### Antibodies

Three different anti-CAR sera were employed (ESM Table 2) based on their selective immunoreactivity against the C-terminus (CAR-CT) or regions within the ECD (CAR-ECD and CAR-RmcB) of the protein, respectively, and validated as described below and in ESM Fig. 1. These allowed the various isoforms of CAR to be distinguished, as illustrated in Fig. 1b. All other antisera are described in ESM Table 2.

### Mutagenesis

Site-directed mutagenesis was performed to remove the final three amino acids (SIV) of CAR-SIV to examine the specificity of the CAR-CT antiserum (ESM Methods).

### Western blotting

The production of CAR in human islets and EndoC-βH1 cells and the specificity of the CAR-CT and CAR-RmcB antisera were assessed using western blotting (ESM Methods).

### Flow cytometry

The specificity of the CAR-CT and CAR-RmcB antisera was assessed using flow cytometry (ESM Methods).

### Immunohistochemistry

This was performed using a standard immunoperoxidase approach [21]. Bright-field image acquisition was performed using a Nikon 50i Microscope fitted with a DS-Fi camera and a DSL2 camera control unit (Nikon, Kingston Upon Thames, UK). Antibody details and conditions are described in ESM Table 2.

### Immunofluorescence

To examine multiple antigens within the same section, FFPE samples were probed in a sequential manner with up to three different antibodies (ESM Table 2) [22]. Pancreas sections were initially subjected to heat-induced epitope retrieval in 10 mmol/l citrate buffer (pH 6), and relevant antigen–antibody complexes were detected using secondary antibodies conjugated with fluorescent dyes (Alexa Fluor anti-mouse 488, anti-rabbit 555, and anti-guinea pig 568 or 647; Invitrogen, Paisley, UK). Cell nuclei were stained with DAPI. After mounting, images were captured either with a Leica AF6000 microscope (Leica, Milton Keynes, UK), then were processed using the standard LAS X Leica software platform (Version 3.3.0.16799), or with a Leica SP8 confocal microscope and Hyvolution2 deconvolution software (Scientific Volume Imaging, Hilversum, the Netherlands). Co-localisation analysis was undertaken with a JACoP plugin from Image J, version 1.48 Java 1.6.0 _20 (https://imagej.nih.gov/ij/plugins/track/jacop2.html).

### Quantitative RT-PCR/semi-quantitative RT-PCR

The relative expression levels of the CAR-SIV, CAR-TVV, CAR4/7, CAR3/7 and CAR2/7 isoforms in isolated human islets and laser capture microdissected (LCM) islets were determined using quantitative (q)RT-PCR (ESM Methods).

### RNAseq

RNA sequencing (RNAseq) was performed using islets obtained from normoglycaemic human islet donors or EndoC-βH1 cells as previously described [23]. Genes and transcripts were assigned a relative coverage rate as measured in reads per kilobase of exon model per million mapped reads and compared with 15 other normal human tissues, analysed by RNAseq and deposited at the Illumina BodyMap 2.0 dataset (GEO accession number GSE30611), accessed on 9 August 2017.

### Cryo-immune electron microscopy

We used cryoimmune electron microscopy to assess and quantify the localisation of CAR-SIV, zinc transporter protein 8 (ZnT8), insulin and proinsulin in human pancreas samples as described previously [24] and in the ESM Methods.

### Co-immunoprecipitation of PICK1 with CAR

This was performed in EndoC-βH1 cells and human islets as described in ESM Methods.

### Statistical analysis

Immunofluorescence images of islets were selected randomly from stained pancreas sections. The Pearson correlation coefficient was used to estimate the co-localisation between proteins using the JACoP plugin from Image J. Values for the Pearson correlation coefficient ranged from 0 for no correlation, to 1 for a positive correlation. GraphPad Prism 5.04 (La Jolla, CA, USA) was employed for all statistical analysis, and data are expressed as means ±SEM. Statistical significance was calculated using one-way ANOVA, and the Bonferroni multiple comparison test was used for multiple comparisons. A *p* value <0.05 was considered statistically significant.

## Results

### Differential CAR immunostaining in human pancreas

The availability of antisera directed against different regions of the CAR protein (Fig. 1b) enabled the expression profiles of the two isoforms bearing a transmembrane domain to be studied. The antiserum designated CAR-CT was raised against a peptide containing the C-terminal 35 amino acids of the CAR-SIV protein and required the presence of the triplet “SIV’ sequence at the immediate C-terminus for immunoreactivity. As such, it did not label any other isoform of CAR. This specificity was confirmed by transfection of 1.1B4 cells with constructs encoding either the full-length CAR-SIV or a variant in which the final three amino acids (SIV) had been removed by targeted mutagenesis (ESM Methods). Western blotting and immunofluorescence staining confirmed immunorecognition of the full-length CAR-SIV isoform, but not the truncated form, by the CAR-CT antiserum (ESM Fig. 1).

Two further CAR antibodies, CAR-ECD (Abcam, Cambridge, UK) and RmcB (Merck Millipore, Watford, UK), both of which recognise the ECD of human CAR, were also used. By deploying a panel of approaches (western blotting, immunofluorescence staining of fixed cells, flow cytometry and immunohistochemistry in FFPE sections), it was found that the CAR-ECD antiserum worked effectively in both western blotting and immunohistochemistry, whereas the RmcB clone was most suitable for immunofluorescence staining of fixed cells or flow cytometry. As noted above, both the CAR-ECD and RmcB antibodies recognise multiple isoforms of CAR including CAR-SIV, CAR-TVV and CAR4/7 (ESM Fig. 1b,c).

Use of these differentially specific antisera revealed that various isoforms of CAR are expressed among the exocrine and endocrine compartments of the human pancreas (Fig. 1c,d). Importantly, the CAR-CT antiserum labelled only islet cells (Fig. 1c) whereas use of the CAR-ECD antiserum resulted in labelling of both exocrine and endocrine tissue (Fig. 1d). An identical staining pattern was confirmed in a total of eight pancreases from non-diabetic donors (within the Exeter and nPOD Biobanks), ranging in age from 4 weeks to 59 years (ESM Table 1).

### Confirmation of SIV isoform expression by qRT-PCR in human islets

**Fig. 2:**
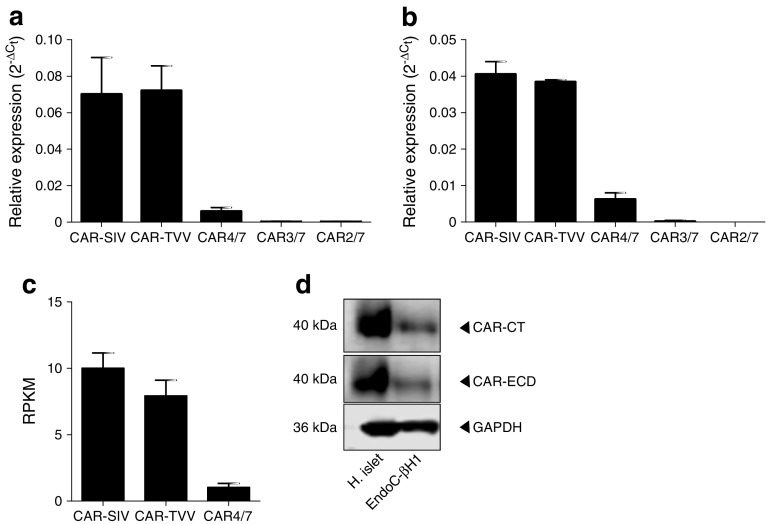
Confirmation of CAR-SIV isoform expression in human islets. qRT-PCR analysis of *CXADR* isoform expression in (**a**) isolated human islets (*n* = 5 individuals) and (**b**) LCM human islets (*n* = 2 individuals) demonstrates that the SIV and TVV isoforms are highly expressed, CAR4/7 is present at low levels, and CAR3/7 and CAR2/7 are barely detectable. Data were normalised to the relative expression of three housekeeping genes, β-actin, *GAPDH* and *B2M*. Relative expression is presented as the mean ± SEM. (**c**) RNAseq data showing CAR isoform expression in islets from five normoglycaemic individuals (mean ± SEM) [23]. RPKM, reads per kilobase of exon model per million mapped reads. (**d**) Confirmation of CAR-SIV protein expression in isolated human islets and EndoC-βH1 cells as assessed by western blotting using CAR-CT and CAR-ECD antisera and loading control glyceraldehyde 3-phosphate dehydrogenase (GAPDH). H., human

Isoform-specific primers were used to analyse *CXADR* isoform expression (ESM Table 3) by qRT-PCR in RNA extracted from isolated human islets (ESM Table 4). This revealed that transcripts encoding the CAR-SIV and CAR-TVV isoforms were expressed at the highest levels, while the soluble CAR4/7 isoform was less abundant (Fig. 2a). The two shorter isoforms CAR3/7 and CAR2/7 were barely detectable. These results were confirmed in a second set of independent samples in which RNA was isolated from LCM islets (Fig. 2b; ESM Table 5). This was further confirmed by RNAseq analysis of isolated human islets (Fig. 2c). Importantly, CAR-SIV was enriched threefold compared with CAR-TVV in the human pancreatic beta cell line EndoC-βH1 (ESM Fig. 2a; ESM Methods) [25], and semi-quantitative RT-PCR analysis of RNA extracted from two highly purified preparations of human islets revealed that the expression of CAR-SIV was greater than that of CAR-TVV (ESM Fig. 2b). Finally, the presence of the SIV isoform was verified by western blotting in isolated human islets and EndoC-βH1 cell using the CAR-CT and CAR-ECD antisera (Fig. 2d).

### The SIV isoform is expressed preferentially in human beta cells

**Fig. 3:**
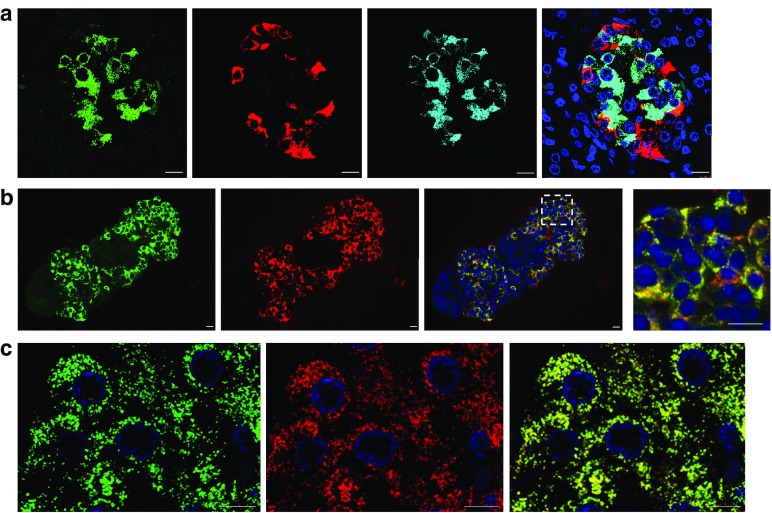
The SIV isoform of CAR is expressed in pancreatic beta cells. (**a**) Representative immunofluorescence staining of the CAR-SIV isoform (CAR-CT antibody; green), insulin (light blue), glucagon (red) and DAPI (dark blue) in an islet from a non-diabetic human pancreas. Scale bars, 10 μm. (**b**) CAR-SIV isoform staining in FFPE isolated human islets: CAR-SIV (CAR-CT; green) and insulin (red) and DAPI (dark blue). The enlarged region (from the area outlined by the dashed white box) demonstrates co-localisation of CAR-CT and insulin staining (yellow). Scale bars, 10 μm. (**c**) Granular distribution of CAR-SIV (green) and co-localisation with insulin (red) and DAPI (dark blue) in the islet of a non-diabetic pancreas. Scale bars, 5 μm. These results are representative of findings in the pancreases of 15 non-diabetic individuals (ESM Table 1)

To determine whether the SIV isoform of CAR is preferentially localised to a specific endocrine cell subset, combined immunofluorescence staining using anti-CAR-CT, insulin and glucagon was performed. This revealed that expression of the CAR-SIV isoform was restricted solely to beta cells in human pancreas sections (Fig. 3a) and isolated human islets (Fig. 3b). Somewhat surprisingly, rather than being localised to the cell surface, the CAR-SIV isoform was distributed mainly within the cytoplasm of beta cells. Confocal microscopy revealed that CAR-SIV displayed a punctate distribution and that it specifically co-localised with insulin, suggesting a possible association with secretory granules (Fig. 3c). A similar pattern of punctate immuno-co-localisation with insulin was observed when using an alternative CAR antiserum (CAR-ECD; data not shown).

To explore these relationships further, the expression of CAR-SIV was investigated in the pancreases of a series of people with type 1 diabetes (ten individuals; 3–42 years of age; ESM Table 1, ESM Fig. 3) and islet autoantibody-positive non-diabetic individuals (two participants, aged 18 and 37 years, respectively). The samples from individuals with type 1 diabetes contained both insulin-deficient islets and insulin-containing islets. CAR-SIV expression was absent from the insulin-deficient islets, but was clearly visible in residual insulin-containing islets. Neither the subcellular distribution nor the staining intensity differed between the type 1 diabetic, autoantibody-positive non-diabetic and non-diabetic islets (ESM Fig. 3 and data not shown). Finally, by applying the CAR-CT antibody to a human tissue microarray (gift from Alan Foulis; University of Glasgow, UK), it was confirmed that the SIV isoform could be variously found at the surface membrane (testes and bladder small cell carcinoma) and/or in cytoplasmic regions (stomach and islets) within normal and cancerous tissues (ESM Fig. 4). RNAseq data supported this expression profile (ESM Fig. 4).

### CAR-SIV co-localises with secretory granule proteins in beta cells

**Fig. 4:**
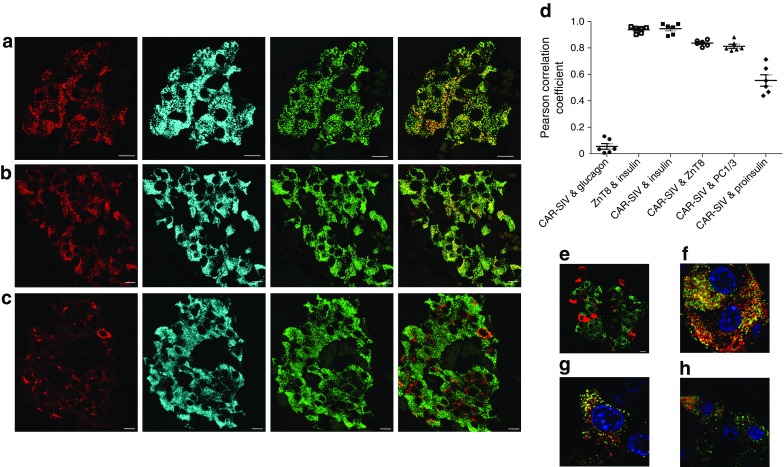
CAR-SIV co-localisation with multiple insulin granule proteins within the beta cell. Representative immunofluorescence staining of the CAR-SIV isoform (CAR-CT antibody; green) and insulin (blue) in relation to insulin secretory granule proteins (red): (**a**) ZnT8, (**b**) PC1/3 and (**c**) proinsulin. (**d**) Pearson correlation coefficient demonstrating the association between CAR-SIV and glucagon, insulin, ZnT8, PC1/3 and proinsulin, and between ZnT8 and insulin. Each data point represents a single islet, and two islets were assessed per case from each of three independent samples. (**e**–**h**) Representative higher magnification images of the CAR-SIV isoform (CAR-CT antibody; green) with (**e**) glucagon, (**f**) ZnT8, (**g**) PC1/3 and (**h**) proinsulin in red. DAPI is shown in dark blue. No association was observed between the CAR-SIV isoform (CAR-CT antibody; green) and glucagon (red) (**e**). Scale, bars 10 μm

In order to verify the localisation of CAR-SIV in beta cells, confocal co-immunofluorescence studies were performed to localise certain other proteins (ZnT8, Fig. 4a; prohormone convertase 1/3 [PC1/3], Fig. 4b; and proinsulin, Fig. 4c) known to be present in secretory granules. Calculation of the Pearson correlation coefficient confirmed that insulin strongly associated with ZnT8 (0.94 ± 0.01; Fig. 4d). Importantly, CAR-SIV also correlated strongly with insulin (0.95 ± 0.02; Fig. 4d) and PC1/3 (0.81 ± 0.02; Fig. 4d). In contrast, CAR-SIV did not associate with glucagon (0.05 ± 0.02; Fig. 4d,e) and was more weakly associated with proinsulin (0.55 ± 0.04; Fig. 4d). High-resolution confocal microscopy confirmed the co-localisation of CAR-SIV with ZnT8 (Fig. 4f) and PC1/3 (Fig. 4g), and showed that CAR-SIV and proinsulin were less frequently co-localised (Fig. 4h). Using a more sophisticated reciprocal analysis, it was confirmed that the majority of CAR-SIV co-localised with insulin (Manders co-localisation coefficient [MCC] 0.914 ± 0.016) and that this was also true in reverse (i.e. the proportion of insulin co-localising with CAR-SIV was high (MCC 0.912 ± 0.028; ESM Fig. 5).

By contrast, although a large proportion of total proinsulin co-localised with CAR-SIV (MCC 0.708 ± 0.082), this did not hold in reverse (CAR-SIV:proinsulin, MCC 0.211 ± 0.042) because little proinsulin escaped into mature secretory granules. Together, these findings suggest that CAR-SIV is present in both immature and mature insulin secretory granules in human beta cells.

### Cryo-immune electron microscopy confirms that CAR-SIV is localised to insulin secretory granules

**Fig. 5:**
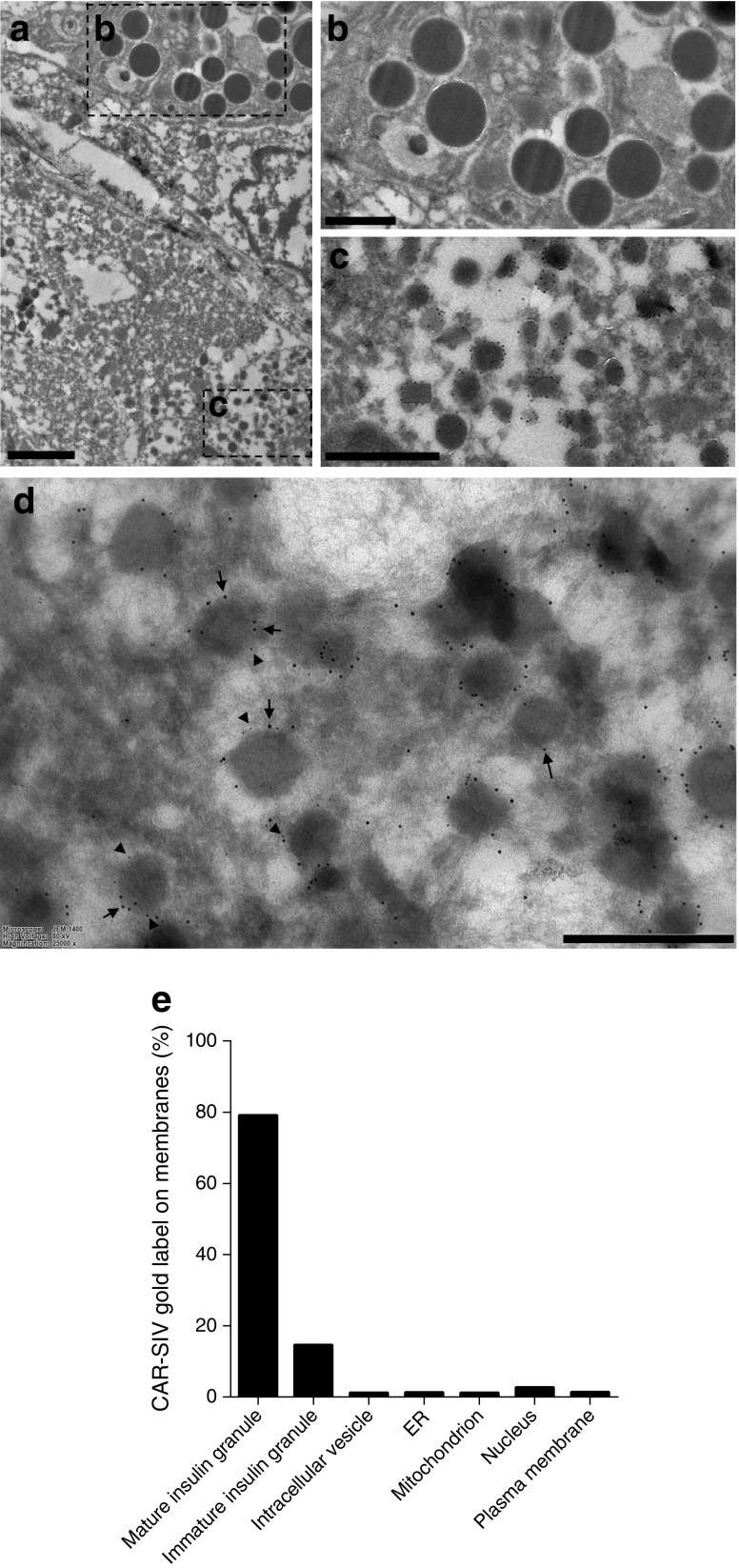
Cryoimmune electron microscopy. Immunogold labelling of CAR-SIV (10 nm gold particles) and ZnT8 (5 nm gold particles) in thin frozen sections of human pancreas tissue. (**a**) The low-magnification image demonstrates the presence of granules in acinar cells and in islet cells. (**b**, **c**) The higher magnification images (of the areas outlined by the dashed black boxes in **a**) reveal a lack of CAR-SIV labelling of acinar cell granules (**b**), but positive CAR-SIV labelling in beta cell granules (**c**). (**d**) A higher resolution, magnified image confirms that the labelling of CAR-SIV (10 nm gold; black arrows) and ZnT8 (5 nm gold; black arrowheads) surrounds the beta cell granules. Scale bars, 2 μm (**a**), 1 μm (**b**, **c**), and 500 nm (**d**). (**e**) Relative distribution of CAR-SIV in organelles based on quantification using line intersection counting. ER, endoplasmic reticulum

To confirm more directly that the CAR-SIV isoform localises to insulin secretory granules, immunogold labelling was performed on thin frozen sections using the post-embedding Tokuyasu method [24] with antisera against CAR-SIV (10 nm gold particles) and ZnT8 (5 nm gold particles). This revealed that both antisera localise to secretory granules in human pancreas sections (Fig. 5); the characteristic electron-dense appearance of the granule cores implies that they contain insulin. This was verified by immunostaining with anti-insulin (ESM Fig. 6). By contrast, gold particles were not concentrated in the secretory granules of exocrine cells. Immunogold labelling of insulin secretory granules with the CAR-CT antibody demonstrated that localisation was least abundant in the centre of the granule cores and preferentially displayed at their periphery (Fig. 5c,d). Labelling of ZnT8 showed a similar distribution.

Quantification of 841 CAR-SIV immunogold particles from 21 different micrographs, across 1291 different membrane intersections, revealed that CAR-SIV was most abundant on mature insulin secretory granules (79%). CAR-SIV was also observed in immature insulin granules (14.4%), but was rarely observed in beta cell nuclei (2.5%), mitochondria (1.0%) or endoplasmic reticulum (1.1%) or on the plasma membrane (1.2%; Fig. 5b,e). Of note, uranyl acetate yields a ‘negative contrast’ for organelle membranes in the staining method employed, and as a consequence the appearance of the secretory granules differs from that seen with osmium labelling.

To further confirm the presence of CAR-SIV in the insulin granules at different stages of granule maturation, immunogold labelling of normal pancreas sections to detect proinsulin (20 nm gold particles), CAR-SIV (10 nm gold particles) and insulin (5 nm gold particles) was performed (ESM Fig. 6). This revealed the presence of CAR-SIV in immature granules, defined as proinsulin and CAR-SIV positive (ESM Fig. 6c); maturing granules, defined as proinsulin, insulin and CAR-SIV positive (ESM Fig. 6d); and mature granules, defined as insulin and CAR-SIV positive (ESM Fig. 6e). Examination of 122 granules containing CAR-SIV revealed that six (4.9%) were also positive for proinsulin (immature granules); 30 (24.6%) were also positive for proinsulin and insulin (maturing granules), and the majority 86 (70.5%) were positive for insulin (mature granules). Taken together, these results suggest that CAR-SIV is present within the granule membrane at all stages of granule maturation.

### CAR-SIV co-localises with PICK1 in insulin secretory granules

**Fig. 6:**
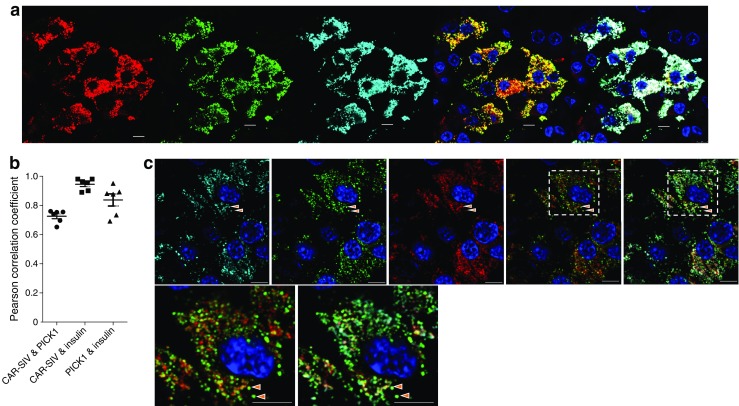
CAR-SIV co-localisation with PICK1 in the beta cell. (**a**) Representative immunofluorescence staining of PICK1 (red), CAR-SIV isoform (CAR-CT antibody; green), insulin (light blue) and DAPI (dark blue) in non-diabetic human pancreas. Overlay of CAR-SIV and PICK1 (yellow), and CAR-SIV, PICK1 and insulin (white). (**b**) Pearson correlation coefficient demonstrating the extent of co-localisation of CAR-SIV with PICK1, CAR-SIV with insulin, and PICK1 with insulin. (**c**) Hyvolution imaging demonstrates a close association of the CAR-SIV isoform (CAR-CT antibody; green) and PICK1 (red) in the insulin granule (light blue) of a non-diabetic pancreas. Granules positive for insulin, PICK1 and CAR-SIV are indicated by the orange arrows. Scale bars, 5 μm

PICK1 may play an important role in insulin granule trafficking and maturation [18], and the SIV isoform of CAR, but not the TVV isoform, selectively interacts with PICK1 in other cell types [13, 15]. To assess whether CAR-SIV co-localises with PICK1 in human beta cells, further confocal co-immunofluorescence studies (Fig. 6) and immunoprecipitation (ESM Fig. 7) studies were performed. PICK1 was readily detected in islet endocrine cells (in both beta and non-beta cells; Fig. 6a) and, importantly, it co-localised with CAR-SIV in beta cells (Fig. 6a) and was co-immunoprecipitated with CAR from EndoC-βH1 cells and human islets (ESM Fig. 7). Pearson’s correlation analysis of the immunofluorescence signals (Fig. 6b) confirmed a strong association between PICK1 and both CAR-SIV (0.73 ± 0.02) and insulin (0.84 ± 0.04).

To examine the co-localisation of PICK1, CAR-SIV and insulin in more detail, confocal microscopy coupled with Hyvolution software was employed to provide improved resolution. This confirmed the intimate association between PICK1 and the SIV isoform of CAR within insulin secretory granules (Fig. 6c). PICK1 was also observed in association with the secretory granules of other islet non-beta endocrine cells, but CAR-SIV was not detected in those cells (Fig. 6).

## Discussion

The present study reveals that the SIV isoform of CAR is expressed selectively by beta cells in the human pancreas. This finding was confirmed at both the RNA and protein levels. We also discovered that the subcellular localisation of CAR-SIV was atypical: the protein was found mainly within the cytoplasmic domain of beta cells rather than at the cell surface.

We used both immunological and molecular biological approaches to investigate CAR expression in human islet cells, and the results were concordant. Thus, analysis of RNA extracted either from isolated human islets or laser capture microdissected islets revealed that two major isoforms of CAR, CAR-SIV and CAR-TVV, were present. Both of these contain a single transmembrane domain, implying that they could each be localised within defined, membrane-limited, compartments in the islet cells, as in other cell types [13]. The results favoured a preponderance of the CAR-SIV isoform in islets, and immunohistochemical analysis confirmed abundant production of CAR-SIV protein in human islet cells, contrasting with its absence from the exocrine pancreas. Taken as a whole, the results suggest that the major isoform of CAR present in human islets is CAR-SIV, although CAR-TVV is also present. CAR-SIV is expressed preferentially in beta cells, since we were unable to find evidence for its expression in ‘non-beta’ islet cells. By contrast, use of less selective antisera suggested that additional isoforms of CAR (including CAR-TVV) may be present among the other non-beta endocrine cells. This would be consistent with the analysis of RNA expression.

The primary subcellular localisation of CAR in beta cells has not been extensively addressed in previous work [3, 20] but, despite this, in other cell types a consensus has emerged that this protein is often localised within tight junctional complexes at the plasma membrane [13, 14, 26]. As such, CAR would be expected to be present at the cell surface, where it could fulfil a secondary (presumably subverted) role as a vehicle for viral entry. Thus, the present demonstration that CAR-SIV is present in human beta cells is consistent with the known sensitivity of these cells to infection by various enterovirus serotypes. However, the intriguing discovery that this isoform was localised primarily at an intracellular site in beta cells suggests a more complex scenario (and a different physiological role) compared with other cells in which CAR resides mainly on the plasma membrane.

High-resolution confocal microscopic analysis was used to examine in more detail the unexpected subcellular localisation of CAR-SIV in beta cells. This revealed a punctate, cytoplasmic immunolabelling pattern for CAR-SIV, consistent with its distribution in a distinct intracellular organelle compartment. Additional studies demonstrated that this immunolabelling profile correlated with that of insulin, as well as with two additional secretory granule proteins, ZnT8 and PC1/3, thereby implying a localisation within beta cell insulin secretory granules. Direct confirmation of this was provided by cryoimmune electron microscopy studies in which immunogold methods allowed the visualisation of CAR-SIV, principally in association with the dense-core granules characteristically found in beta cells. By contrast, no labelling was seen in the equivalent secretory granules found in adjacent exocrine cells, implying that CAR-SIV is not absolutely required for secretory granule biogenesis in all cell types.

Importantly, and consistent with results obtained in other cells [18], we also noted that CAR-SIV co-localised with, and could be immunoprecipitated with, the PDZ domain protein PICK1 in beta cells. PICK1 plays a specific role in insulin secretory granule maturation [18, 27], and it is conceivable that CAR-SIV serves as a selective binding partner for PICK1 in beta cells, thereby concentrating the two proteins within maturing secretory granules. Consistent with this hypothesis, we found by immunofluorescence analysis that the SIV isoform of CAR is much more strongly associated with mature insulin than with proinsulin, suggesting that CAR (and PICK1) may become concentrated in secretory granules as these mature beyond their emergence from the *trans*-Golgi network.

If this model is correct, it has additional important consequences. In particular, the model predicts that the C-terminus of CAR-SIV must face the extragranular environment, since its PDZ-binding domain is located in this region and would only be available to interact with cytoplasmic PICK1 (or other cytoplasmic PDZ-binding proteins) in this orientation. As such, the putative ECD of CAR-SIV would then face the lumen of the granule, with the single transmembrane region serving to anchor the protein in this orientation in the limiting membrane surrounding the granule. Thus, the region of SIV required for the binding of enteroviruses would face the interior of the secretory granule during maturation. It follows from this that the ECD of CAR-SIV would become displayed on the external face of the plasma membrane following the fusion of the secretory granule and plasma membranes during exocytosis.

**Fig. 7:**
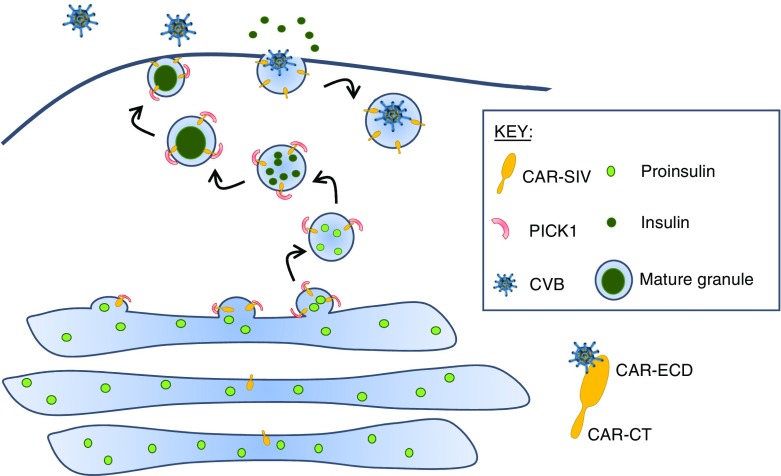
CAR-SIV in beta cells. Our data demonstrate that CAR-SIV is present at high concentrations on the insulin granule and is closely associated with the cytoplasmic protein PICK1. CAR-SIV has previously been shown to interact with PICK1, which is proposed to have a role in the budding and maturation of vesicles from the *trans*-Golgi network. We predict that the C-terminus of CAR-SIV faces the extragranular/cytoplasmic environment since its PDZ-binding domain would be available to interact with cytoplasmic PICK1 only in this orientation. We propose that CAR-SIV, through its interaction with PICK1, could therefore play a hitherto unsuspected role in the maturation and trafficking of the insulin granule. Importantly, when considering the orientation of CAR-SIV in this model, the putative ECD of CAR-SIV, which is required for the binding of enteroviruses, faces the interior of the secretory granule during its maturation. This suggests that, as the insulin granule fuses with the plasma membrane during insulin exocytosis, the ECD of CAR-SIV becomes displayed on the external face of the plasma membrane and is then able to bind to enteroviruses that use this receptor, for example CVBs. During the subsequent endocytosis of the granule, for recycling, the virus would be transported inside the cell, where it could initiate a productive infection

These considerations are summarised in Fig. 7 and suggest that enteroviral entry into beta cells may be facilitated under conditions in which the rate of secretory granule exocytosis is high (and granule membrane recycling rates are correspondingly elevated). Thus, CAR-SIV appears to be configured in beta cells such that it can interact with PDZ-binding proteins during secretory granule maturation, and its virus-binding domain becomes exposed to the extracellular environment during exocytosis. Of note, most of the autoantigens in type 1 diabetes are expressed in the insulin granule [28] and the preferential localisation of viral receptors in the granule indicates a potential mechanism by which CVB infection may modify the processing of granule proteins, to promote the generation of autoantigens.

In support of our conclusions, Ylipaasto et al [3] demonstrated that the pre-treatment of human islets with a blocking antibody directed solely against externally oriented CAR attenuated infection with Coxsackie viruses. When coupled with our finding that the majority of CAR is located intracellularly in islet cells, this suggests that externalisation of the protein occurs to mediate viral entry. Moreover, we also note Hodik et al’s [29] demonstration that enterovirus replication complexes and viral particle lattices are present on or near to insulin granules in CVB acutely infected beta cells. Thus, this weight of evidence is strongly supportive of our hypothesis and deserves further study.

In summary, we show that human beta cells express the SIV isoform of CAR in the insulin granules. We propose that the biochemical properties of CAR-SIV that confer the physiological importance of this protein within beta cells may also represent an ‘Achilles heel’ by which the entry and replication of enteroviruses is facilitated.

## Electronic supplementary material

ESM (PDF 962 kb)

## Data Availability

The datasets generated during and/or analysed during the current study are available from the corresponding author on reasonable request.

## Abbreviations

CAR: Coxsackie and adenovirus receptor
CAR-CT: Coxsackie and adenovirus receptor C-terminus
CVB: Coxsackievirus B
ECD: Extracellular domain
FFPE: Formalin-fixed, paraffin-embedded
LCM: Laser capture microdissected
MCC: Manders co-localisation coefficient
PC1/3: Prohormone convertase 1/3
PICK1: Protein interacting with C-kinase 1
RNAseq: RNA sequencing
ZnT8: Zinc transporter protein 8
