# Supplementary material for: Unexpected subcellular distribution of a specific isoform of the Coxsackie and adenovirus receptor, CAR-SIV, in human pancreatic beta cells
Source: Diabetologia. 2018 Aug 3;61(11):2344–55. doi: 10.1007/s00125-018-4704-1 (PMC6182664; doi:10.1007/s00125-018-4704-1)
Supplement: Supplementary file 1 — (PDF 962 kb) [file 125_2018_4704_MOESM1_ESM.pdf]

ESM METHODS:

Cell culture

The human beta cell line EndoC-βH1 (kindly provided by Dr. R. Scharfmann, University of Paris, France (1) was cultured in Matrigel-fibronectin-coated plates as described (2). Cells were cultured in DMEM containing 5.5mmol/l glucose, 2% bovine serum albumin (fraction V), 50µmol/l β-mercaptoethanol, 5.5µg/ml transferrin, 6.7ng/ml sodium selenite, 10mM nicotinamide, penicillin (100units/ml) and streptomycin (100µg/ml). Cells were seeded at 1.75 x10^6/ml on an extracellular matrix (ECM) with fibronectin coated T25 flasks and were sub-cultured every 7-9 days. Half of the culture medium was replaced every 3-4 days. Human 1.1B4 cells which were a gift from Prof. Peter Flatt (3) were cultured in RPMI 1640 medium containing 11.1mM glucose and supplemented with 10% fetal bovine serum, 2mmol/l L-Glutamine, 100U/ml penicillin and 100µg/ml streptomycin.1.1B4 cells were sub-cultured upon reaching 80% confluence and both cell lines were maintained at 37^0C, 100% humidity and 5% CO_2. All cells were mycoplasma negative.

Mutagenesis

A pCMV6 vector containing the SIV isoform of the human *CXADR* gene with a Myc-DDK tag was purchased (ORIGENE Rockville, MD, USA).The $Q5^R$ site directed mutagenesis kit was used according to the manufacturer's instruction (New England Biolabs, Hitchin, UK) to produce truncated variants of the protein encoded by the cDNA. Primers were synthesized to generate the SIV isoform without a Myc-DDK tag (F: GTCTATAGTATAGCGTACGCGGCC; R: CCATCCTTGCTCTGTGCT) or without the final three amino acids of the isoform (F: GACCAAGGATTAGTCTATAGTAACG; R:

TGTGCTGGAATCATCACAG). All variants were fully sequenced to confirm the success of mutagenesis (Source Bioscience, Nottingham, UK).

Western blotting

Cells were collected and lysed in buffer (20mmol/l Tris, 150mmol/l Nacl, 1mM EDTA and 1% Triton-X including protease and phosphatase inhibitors (Sigma, Poole, UK)) on ice for 10mins. Protein lysates was separated from insoluble fraction by centrifugation at 5000rpm for 10mins at 4^0C. Total protein was estimated with the Pierce ™ (BCA) Protein Assay (Thermofisher Scientific, UK) and absorbance was measured at 562nm with a PHERAstar (BMG Labtech). Equal amount of proteins (20-50ug) were adjusted with 25% of 4x lithium dodecyl sulphate (LDS) loading buffer and 10% β-mercaptoethanol prior to denaturing at 70^oC for 10mins. Protein samples were loaded onto a pre-cast 12% Bis-Tris poly-acrylamide gel (Invitrogen) and electrophoresis was carried out using XCell Sure Lock (Invitrogen) at 120V in 20x MOPS SDS running buffer (Invitrogen) for 1hr. Proteins were electro-transferred onto a methanol permeabilized polyvinylidene difluoride (PVDF) membrane using XCell II Blot Module (Invitogen, UK) containing transfer buffer (Glycine, Tris, Methanol, ddH20) at 30V for 2hrs.

Membranes were blocked in 5% skimmed milk (Sigma) in Tris buffered saline with 0.05% Tween 20 (TBST) buffer at room temperature for 1hr. Primary antibodies; anti-CAR-CT (1:1000, ab100811, Abcam), anti-CAR-ECD (1:500, ab180761, Abcam), β-actin (1:10000, A5441, Sigma), anti-PICK1 (1:1000, Abcam ab133773) or GAPDH (1:10000, 60004-1-Ig, Proteintech) were diluted in 5% milk/ TBST and membranes probed overnight at 4^0C, with the exception of β-actin and GAPDH (2hrs at room temperature). The membrane was washed 3x with TBST and probed with a secondary antibody raised against mouse or rabbit conjugated to Alkaline phosphatase (A3562, A3687, Sigma) or Goat anti-rabbit IgG (H+L) highly crossed

absorbed, Alexafluor plus 680 (#A327-34, Thermofisher) in 1% milk/ TBST for 1hr at room temperature. Finally, the membrane was washed 3x with TBST and bands were visualised with a CDP-Star Chemiluminescent substrate (Sigma, C0712) using either a Li-COR C-Digit or Odyssey CLX (Li-COR Biosciences, UK)

Flow cytometry

Cells were trypsinised and re-suspended in FACS buffer (PBS, 2% FBS). For intracellular staining, cells were fixed with 4% paraformaldehyde, washed twice with FACS buffer and incubated with either anti-CAR CT or anti-CAR-RmCB-PE (FCMAB418PE, Merck Millipore) or negative control antibodies. Rabbit Isotype IgG (I-1000 Vector laboratories) or Mouse IgG1k (FCMAB230P, Merck) in 0.3% saponin at 4^0C for 45mins. Cells were then washed in 0.03% saponin in FACS buffer and then analysed immediately on a Flow cytometer (BD Accuri ™ C6 Plus) or if they were not directly conjugated to a fluorochrome, they were incubated with secondary antibody (Alexa-488) for 30mins prior to analysis. For extracellular staining, cells were incubated with either CAR-CT or CAR-Rmcb-PE in FACS buffer at room temperature for 45mins. Cells were then washed in FACS buffer and analysed or incubated with secondary antibodies (Alexa-488) before analysis. Results were confirmed in 3 independent experiments.

Laser capture microdissection of human islets

Laser capture microdissection (LCM) of human islets was performed on OCT frozen sections from pancreas of non-diabetic nPOD donors (6017, 6096; Supplementary Table 4). Briefly, 5µm thick frozen sections were fixed in 70% ethanol for 30 seconds and then dehydrated in 100% ethanol, xylene and then air dried for 5 minutes. An Arcturus Xt microdissection

instrument (Thermofisher, Waltham, MA, USA) was used to capture human islets based on beta-cell autofluorescence. Hs-Caps (Thermofisher) were used to microdissect identified islets. Microdissected islets were then lysed in Arcturus Picopure kit Extraction Buffer for 30 minutes at 42°C and subjected to total RNA extraction using Arcturus Picopure RNA extraction kit following the manufacturer's instructions (Thermofisher).

Real Time RTqPCR

Total RNA from isolated human islets was extracted using miRNeasy mini kit (Qiagen, Hilden, Germany). Briefly, human isolated islets were lysed in Qiazol solution and chloroform was added to separate DNA, RNA and proteins. The aqueous phase was then loaded on to RNA binding columns and eluted in 30μl of nuclease-free water. RNA quality were evaluated using a 2100 Bioanalyzer RNA 6000 Pico kit (Agilent Technologies, Santa Clara, CA, USA) and only samples with RNA Integrity Number (RIN) >5.0 were used for further analyses.

Differential expression of alternatively spliced isoforms of the human *CXADR* gene were evaluated using custom designed TaqMan primers and probes (Thermofisher) (ESM Table 3). For human isolated islets (ESM Table 4), 250ng of total RNA was retro-transcribed using Superscript III Reverse Transcriptase kit (Thermofisher) and 10ng of the resultant cDNA were loaded into 2x TaqMan Universal Master Mix, 20x TaqMan gene expression assay buffer and nuclease-free water in a final volume of 20μl. Data were normalised using the expression of β-Actin, GAPDH and β2-microglobulin. Reactions were performed on a Verity Thermal Cycler and ViiA7 Real Time PCR instruments (Thermofisher). Data were analysed and exported using Expression Suite software 1.1 (Thermofisher) and finally elaborated using the $2^{-\Delta Ct}$ method.

For LCM captured islets (ESM Table 5), 1ng of total RNA was retro-transcribed using Superscript III Reverse Transcriptase kit (Thermofisher). The resultant cDNA was pre-amplified using 0.2x Tris-EDTA-diluted TaqMan assays pool (designed to amplify *CXADR* isoforms plus 3 housekeeping genes (β-Actin, GAPDH, β2-microglobulin)) and 2x Preamp Master Mix in a final volume of 50µl. The pre-amplification reaction was diluted in Tris-EDTA and 5µl of each pre-amplified cDNAs were used in a Real-Time PCR reaction in a final volume of 20µl.

Semi-quantitative RT-PCR

Total RNA was isolated from human islets (ESM Table 4) using RNeasy mini kits (Qiagen, Manchester, UK). 1µg of RNA was reverse transcribed (Promega, Madison,WI, USA) to synthesize cDNA. Specific primers were designed to selectively target the two *CXADR* transmembrane isoforms; CAR-SIV (F: GGAAGTTCATCACGATATCAG; R:AATCATCACAGGAATCGCAC), CAR-TVV (F: GGAAGTTCATCACGATATCAG; R: TTCCATCAGTCTTGTAAGGG). Amplified bands were excised and sequenced (Source Bioscience, Nottingham, UK) to confirm the isoform expression pattern.

Cryo-immune EM

For cryo-immuno-EM human samples were treated as described [24]. Briefly, human pancreas tissue samples (nPOD 6227, 6229 and 6330) were fixed in 4% PFA, cut into small pieces and immersed in 2.3 mol/l sucrose in PBS at 4°C. The samples were placed on metal stubs, frozen in liquid nitrogen and thin cryosections were cut and protected with sucrose/methyl cellulose films. For immunolabeling, the protective layer was first melted in gelatin coated dishes at 37°C and then washed with 0.1% glycine in PBS. Then grids were incubated in PBS containing 1% BSA with appropriate antibodies (ESM Table 2) for 45 minutes at room temperature.

Protein A-gold labelled antibodies (diameters 5, 10 or 20nm; G. Posthuma, Utrecth) diluted in 0.1% BSA in PBS was added onto the grids for 20 min followed by washes with 0.1% BSA/PBS and PBS alone. A bridging antibody was used to link the proinsulin to the 20nm gold particles. In double or triple labelling studies, antibodies were added sequentially with their respective protein-A gold conjugate, between incubations the sections were incubated briefly with 1% glutaraldehyde. The sections were then stained with 2% neutral uranyl acetate in water for 5 min and embedded for 10 min on ice with 2% methylcellulose containing 0.4% uranyl acetate and examined with a Jeol 1400 microscope.

Quantification of CAR-SIV in Cryo-immune EM

Quantification of CAR-CT immuno-gold labelling was performed by conventional line intersection counting from electron micrographs of human pancreas sections labelled with anti-CAR-CT (4). The same array of test lines was overlaid at random positions on all micrographs. Intersections with membrane limited compartments (mature and immature secretory granules, exocrine granules, endoplasmic reticulum, mitochondria, nucleus and plasma membrane) were assessed and the gold particles in these specific intersections counted. 21 micrographs were analysed yielding a total of 841 gold particles and 1291 intersections between membrane compartments. Numbers of gold particles per intersection were calculated and normalized for the abundance of each relevant compartment. Data were then displayed as the percentage of CAR-SIV gold particles in each of the different compartments. For ESM Fig 6, the number of CAR-SIV positive granules that were also labelled with proinsulin and/ or insulin labelled gold particles were counted.

Co-immunoprecipitation of PICK1 with CAR

Whole cell extracts were prepared by lysing 5 x 10^5 EndoC-βH1 cells or human islets with buffer containing 50mmol/l Tris (pH 7.5); 137mmol/l NaCl; 5mmol/l EDTA; 1mmol/l EGTA; 10µg/ml protease inhibitor (Sigma) and 10µg/ml phosphatase inhibitor cocktail 2 & 3 (Sigma, UK). The lysates were incubated with 3µg of anti-CAR clone RmcB or negative control mouse IgG (Dako; X0931) overnight at 4^0C. Protein G Sepharose beads were then added for 4 hours at 4^0C followed by three washes [first - lysis buffer; second -10% lysis buffer in TBS (50mmol/l Tris (pH 7.5) and 137mmol/l NaCl); Third – TBS). Proteins were eluted with 4x LDS and 10% β-mercaptoethanol at 70^0C for 10min. Western blot analysis was then performed as described above.

ESM Table 1: Tissue Samples, Patient Information

Case ID	Case Type	Cohort	Age	Sex	Duration of disease
12425	No diabetes	EADB	Neonate	N/A	
8582	No diabetes	EADB	1	N/A	
150/88	No diabetes	EADB	3	F	
274/91	No diabetes	EADB	6	M	
245/90	No diabetes	EADB	6	M	
8651	No diabetes	EADB	6	N/A	
12229	No diabetes	EADB	10	N/A	
540/91	No diabetes	EADB	11	M	
6099-06	No diabetes	nPOD	14.2	M	
PM146/66	No diabetes	EADB	18	F	
PAN 8	No diabetes	EADB	19	N/A	
PAN 1	No diabetes	EADB	22	N/A	
6160-06	No diabetes	nPOD	22.1	M	
329/72	No diabetes	EADB	24	M	
191/67	No diabetes	EADB	25	M	
PM132/67	No diabetes	EADB	46	M	
330/71	No diabetes	EADB	47	M	
186/74	No diabetes	EADB	55	F	
77/6/87	No diabetes	EADB	57	F	
332/66	No diabetes	EADB	59	M	
6098	No diabetes	nPOD	17.8	M	
6153	No diabetes	nPOD	15.2	M	
6027	AAb+	nPOD	18.8	M	
6167	AAb+	nPOD	37	M	
E236	T1D	EADB	7	F	'Recent'
SC41	T1D	EADB	4	F	3 weeks
11746	T1D	EADB	6	M	<1 week
11713	T1D	EADB	3	M	3mth
E375	T1D	EADB	11	F	1 week
E560	T1D	EADB	42	F	18mths
6041	T1D	nPOD	26.3	M	23y
6087	T1D	nPOD	17.5	M	4y
6113	T1D	nPOD	13.1	F	1.58y
6161	T1D	nPOD	19.2	F	7y

EADB – Exeter Archival Diabetes Biobank; AAb+ - Autoantibody positive no diabetes; N/A – not avaliable

ESM Table 2: Antibody Details and Conditions for IHC/ IF. All antisera were validated with positive and negative controls and the CAR antisera were further validated (ESM Fig 1)

Primary Antibody	Manufacturer and clone	IHC/IF Antigen Retrieval	Conditions and Secondary Detection System
**CAR-CT**	Abcam ab100811<br>Rabbit polyclonal	10mmol/l citrate pH6.0	Dako REAL Envision Detection System or Immunofluorescence staining (1/1000 for 1hr) using anti-rabbit IgG (H+L) Alexa Fluor-conjugated secondary antibodies (1/400 for 1hr)
**CAR-ECD**	Abcam ab180761<br>Rabbit polyclonal	10mmol/l citrate pH6.0	Dako REAL Envision Detection or Immunofluorescence staining (1/200 for 1hr) using anti-rabbit IgG (H+L) Alexa Fluor-conjugated secondary antibodies (1/400 for 1hr)
**CAR RmcB**	Merck #05-644<br>Lot # 2880699<br>Mouse monoclonal	Utilised for ICC and IP	ICC Immunofluorescence staining (1/400 for 1hr) using anti-mouse IgG (H+L) Alexa Fluor-conjugated secondary antibodies (1/400 for 1hr)
**Insulin**	Dako<br>C#A0564<br>Guinea-pig polyclonal	10mmol/l citrate pH6.0	Immunofluorescence staining (1/700 for 1hr) using anti-guinea-pig IgG (H+L) Alexa Fluor-conjugated secondary antibodies (1/400 for 1hr)
**Glucagon**	Abcam<br>C#ab82270<br>Mouse monoclonal	10mmol/l citrate pH6.0	Immunofluorescence staining (1/2000 for 1hr) using anti-mouse IgG (H+L) Alexa Fluor-conjugated secondary antibodies (1/400 for 1hr)

**Proinsulin**	Abcam<br>ab8301<br>Mouse monoclonal	10mmol/l citrate pH6.0	Immunofluorescence staining (1/500 for 1hr) using anti-mouse IgG (H+L) Alexa Fluor-conjugated secondary antibodies (1/400 for 1hr)
**ZnT8**	R & D<br>C#815039<br>Mouse monoclonal	10m mmol/l citrate pH6.0	Immunofluorescence staining (1/63 for 1 hr) using anti-mouse IgG (H+L) Alexa Fluor-conjugated secondary antibodies (1/400 for 1hr)
**PC1/3**	Abcam<br>Ab55543<br>Mouse monoclonal	10m mmol/l citrate pH6.0	Immunofluorescence staining (1/500 for 1hr) using anti-mouse IgG (H+L) Alexa Fluor-conjugated secondary antibodies (1/400 for 1hr)
**PICK1**	Santa Cruz	10m mmol/l Citrate pH6.0	Immunofluorescence staining (1/50 for 1hr) using anti-mouse IgG (H+L) Alexa Fluor-conjugated secondary antibodies (1/400 for 1hr)

ESM Table 3: TaqMan Probe Sequences

**Target sequence**	**Probe ID**	**Taqman Probe Sequence**
CXADR EXON_2_7	AIKAMSZ	ggtggatcaagtgggaagat
CXADR EXON_3_7	AILJKY7	gtagttcttgggaagatgtg
CXADR EXON_4_7	AIMSI5F	catggttagcagggaagatg
CXADR EXON_6_7 (CAR-SIV)	AIN1HBN	cgatatcagggaagatgtgc
CXADR EXON_6_7_8 (CAR-TVV)	AIPAFHV	ccaacatggaaggatattcc

ESM Table 4: Human islet donors, samples used for TaqMan qRT-PCR

**Sample ID**	**Gender**	**Age (y)**	**BMI (Kg/m^2)**
**1**	M	52	34.2
**2**	M	50	27.4
**3**	M	55	28.0
**4**	F	79	23.9
**5**	M	59	26.7

ESM Table 5: Donor information for LCM islet isolation

**Sample ID**	**Gender**	**Age (y)**	**BMI (Kg/m^2)**	**No. Of LCM captured islets**	**RIN Value**
**6017**	F	59	24,8	52	5.1
**6096**	F	16	18,8	39	7.2

References

(1) Ravassard P, Hazhouz Y, Pechberty S, Bricout-Neveu E, Armanet M, Czernichow P, Scharfmann R: A genetically engineered human pancreatic β cell line exhibiting glucose-inducible insulin secretion. The Journal of clinical investigation 2011;121:3589-3597

(2) Brozzi F, Nardelli TR, Lopes M, Millard I, Barthson J, Igoillo-Esteve M, Grieco FA, Villate O, Oliveira JM, Casimir M, Bugliani M, Engin F, Hotamisligil GS, Marchetti P, Eizirik DL: Cytokines induce endoplasmic reticulum stress in human, rat and mouse beta cells via different mechanisms. Diabetologia 2015;58:2307-2316

(3) McCluskey JT, Hamid M, Guo-Parke H, McClenaghan NH, Gomis R, Flatt PR: Development and functional characterization of insulin-releasing human pancreatic beta cell lines produced by electrofusion. Journal of Biological Chemistry 2011;286:21982-21992

(4) Griffiths G (1993) Fine Structure Immunocytochemistry. Heidelberg, Springer. DOI 10.1007/978-3-642-77095-1.

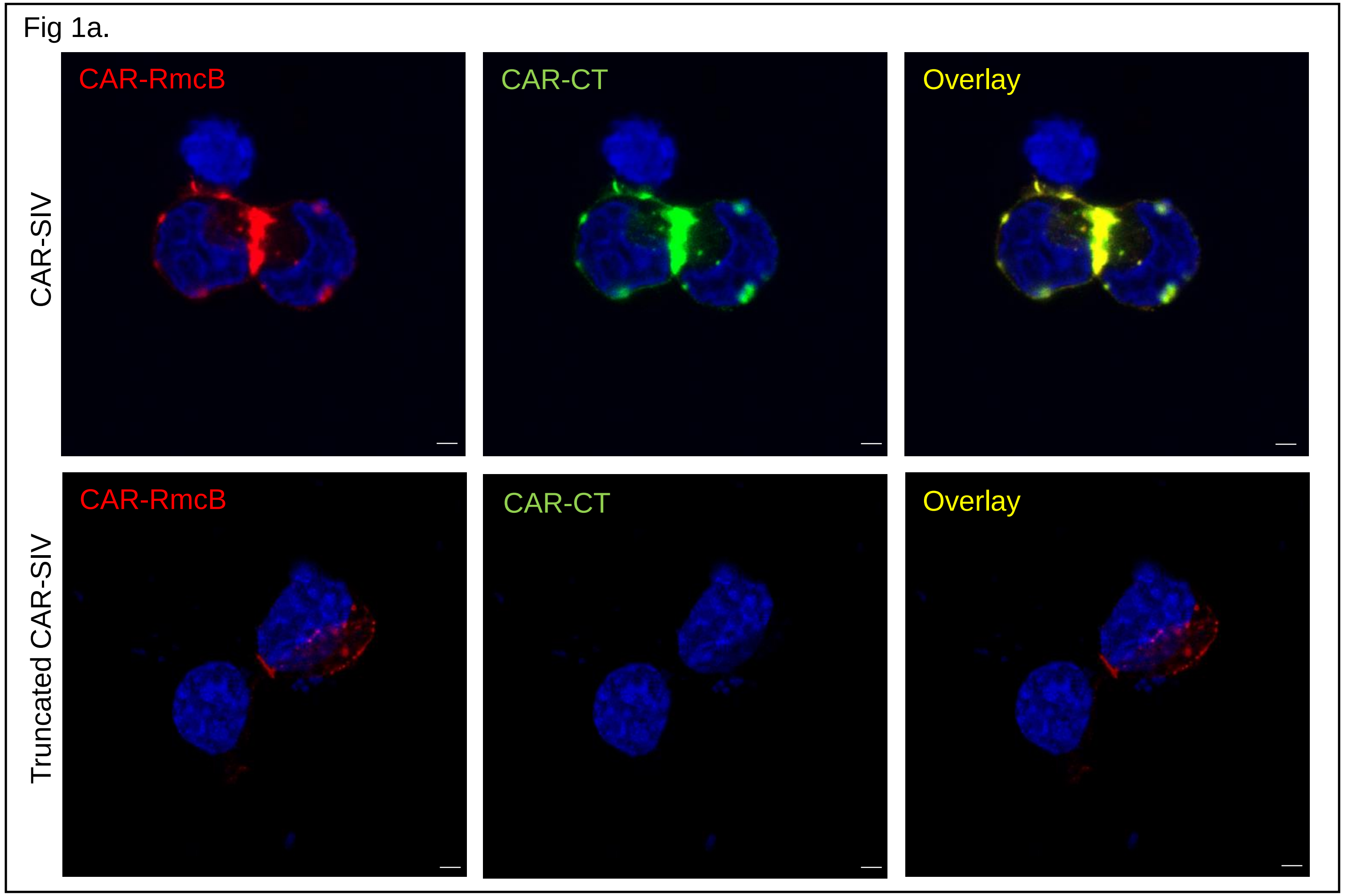

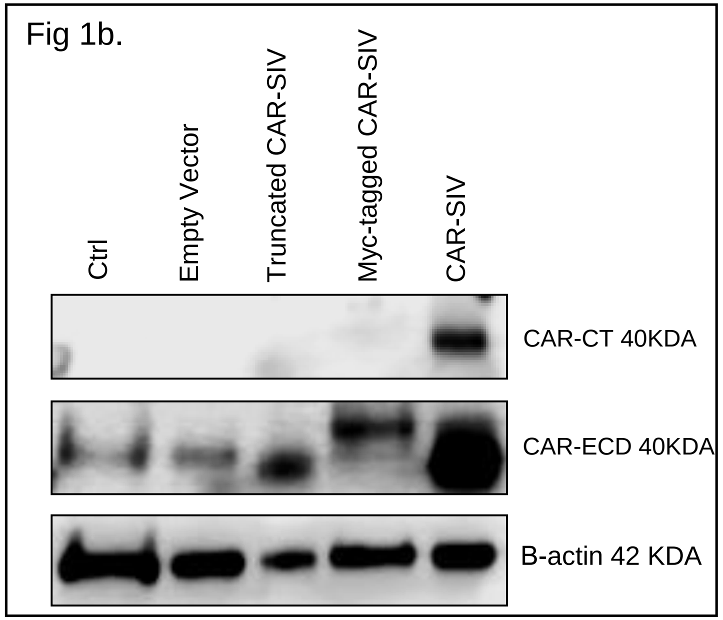

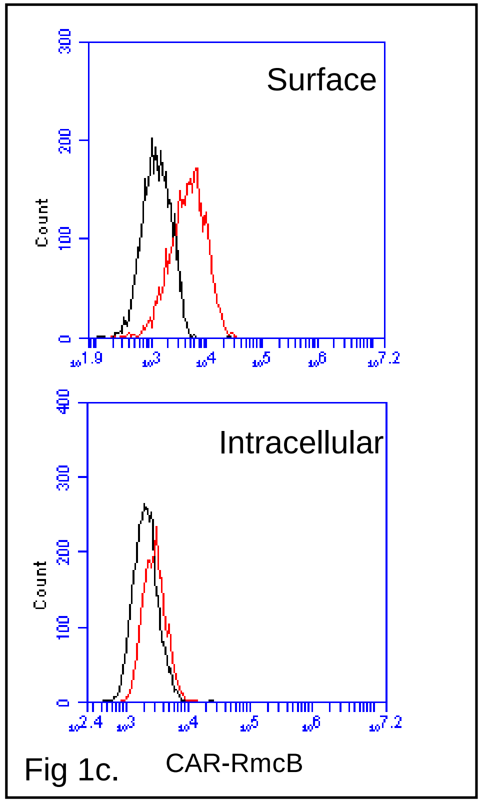

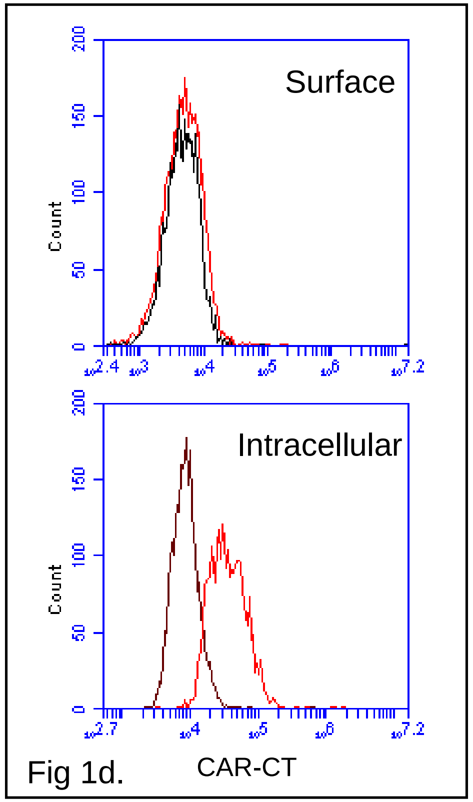

ESM Fig. 1: Analysis of CAR antibody specificity. (a) 1.1B4 cells (see Supplementary methods) were transfected with either the full length CAR-SIV (upper panel) or truncated CAR-SIV (lower panel) and probed with CAR-RmcB (red) or CAR-CT (green) antisera. Scale bar 2.5µm. The full length CAR-SIV is recognised by both the CAR-CT and the CAR-RmcB antisera. In contrast, the truncated CAR-SIV is only recognised by the CAR-RmcB antiserum, demonstrating the absolute requirement for the terminal three amino acids for CAR-CT antiserum binding. **(b)** Western blot analysis confirms that CAR-CT antisera detects only the full length CAR-SIV (40kDa) isoform in transfected 1.1B4 cells. Cells transfected with either the truncated CAR-SIV or the Myc-tagged CAR-SIV (where the Myc-tag immediately at the C teminus appears to hinder antibody access) are not recognised by the CAR-CT antiserum. In contrast the CAR-ECD antibody that detects the ECD of CAR can recognise other CAR isoforms including CAR-TVV (40kDa) visible in control and empty vector cells, as well as the CAR-SIV shown in the truncated CAR-SIV, Myc-tagged CAR-SIV and the full length CAR-SIV transfected cells. Representative flow cytometry plots of **(c)** CAR-RmcB and **(d)** CAR-CT antisera staining of the surface (upper panels) and intracellular expression (lower panels) on EndoC-βH1 cells.

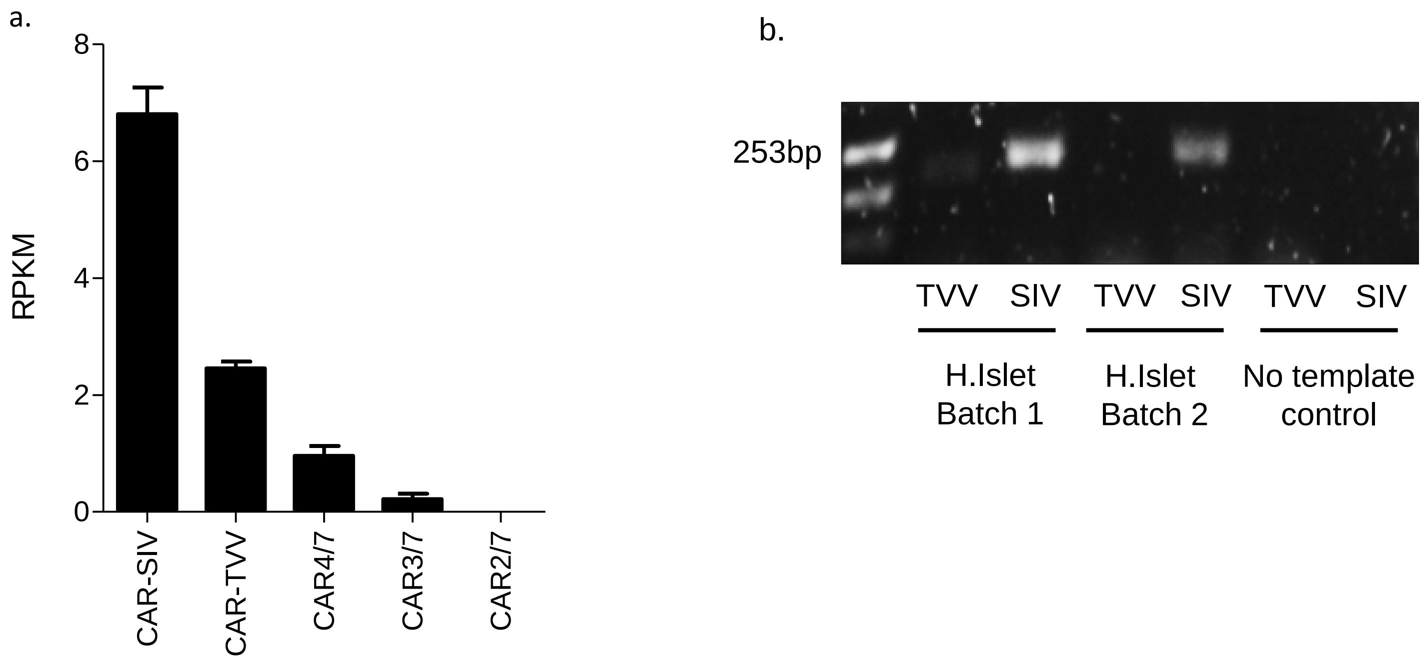

ESM Fig. 2: **(a)** RNAseq analysis of CAR isoform expression in 5 preparations of the human pancreatic beta cell line (ESM Table 4), EndoC-βH1 (Mean±SEM). CAR-SIV is expressed at levels 3-fold higher than CAR-TVV. CAR4/7 and CAR3/7 are present at low levels and CAR2/7 was undetectable. **(b)** Semi-quantitative RT-PCR (30 cycles) analysis using specific CAR-SIV and CAR-TVV primers in RNA extracted from purified human islets, suggests in two independent islet preparations that CAR-SIV is present at a higher abundance than CAR-TVV.

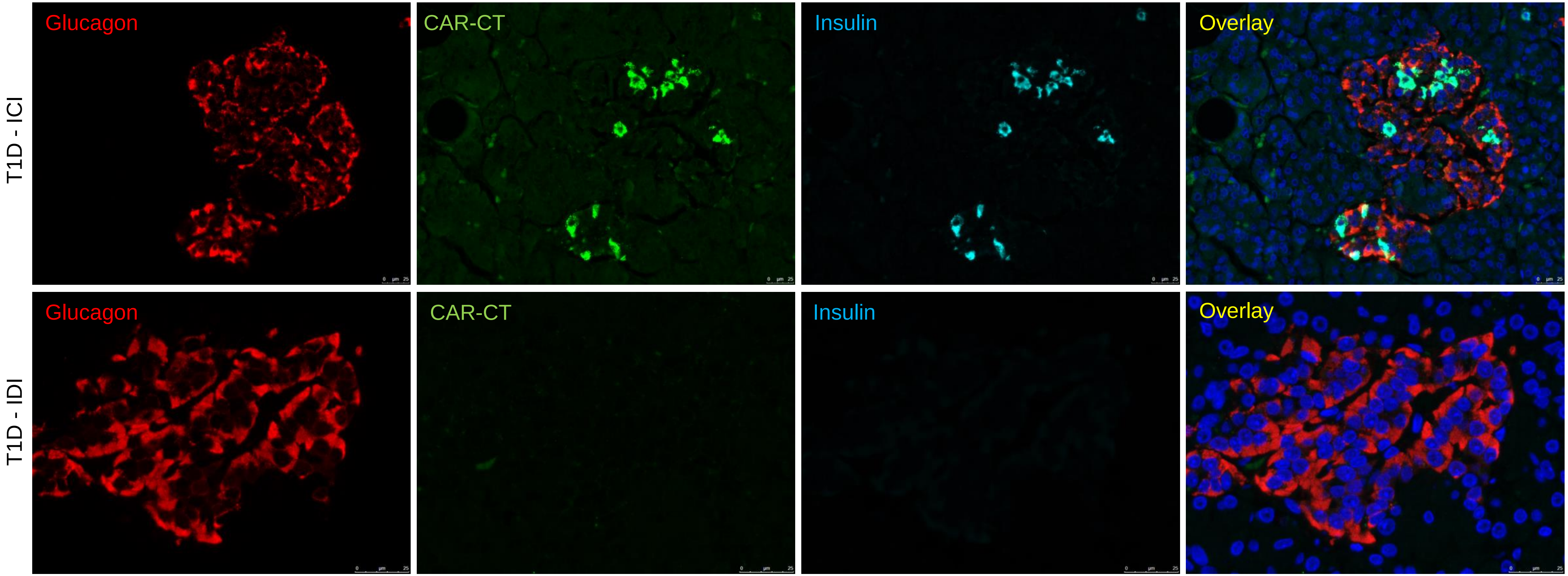

ESM Fig 3: CAR-SIV expression is observed only in the insulin containing islets of individuals with Type 1 diabetes patients and not in insulin deficient islets. Representative immunofluorescence staining of the CAR-SIV isoform (CAR-CT antibody; green) insulin (light blue), glucagon (red) and DAPI (dark blue) in an insulin containing islet (ICI; upper panel) and insulin deficient islet (IDI; lower panel) of a donor with Type 1 diabetes. Scale bar 25µm. These results are representative of findings in the pancreas of 10 Type 1 diabetes donors (ESM Table 1). CAR-SIV is expressed in T1D donor islets but only in beta cells (upper panel). Once the beta cells have been destroyed CAR-SIV expression is lost (lower panel). CAR-SIV expression in beta cells from autoantibody positive non-diabetic donors did not differ from that seen in autoantibody negative donors without diabetes (data not shown).

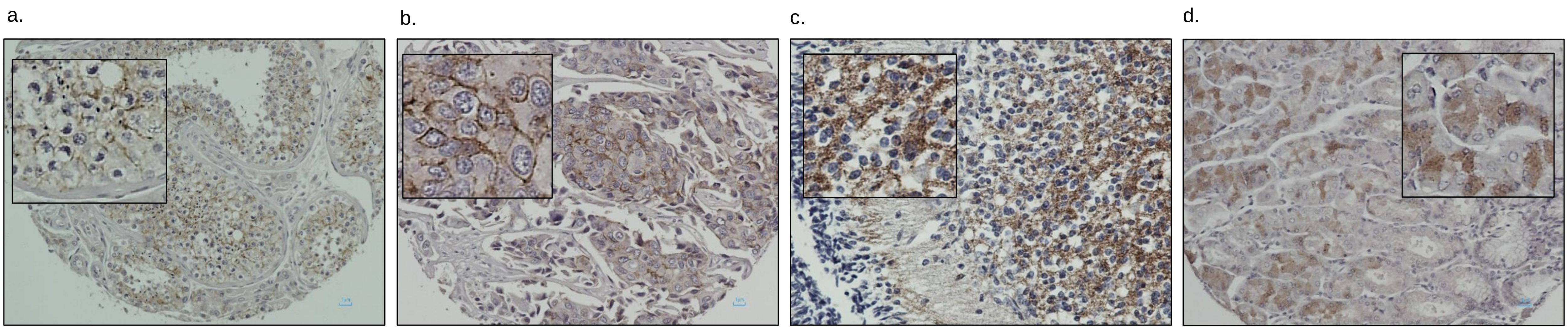

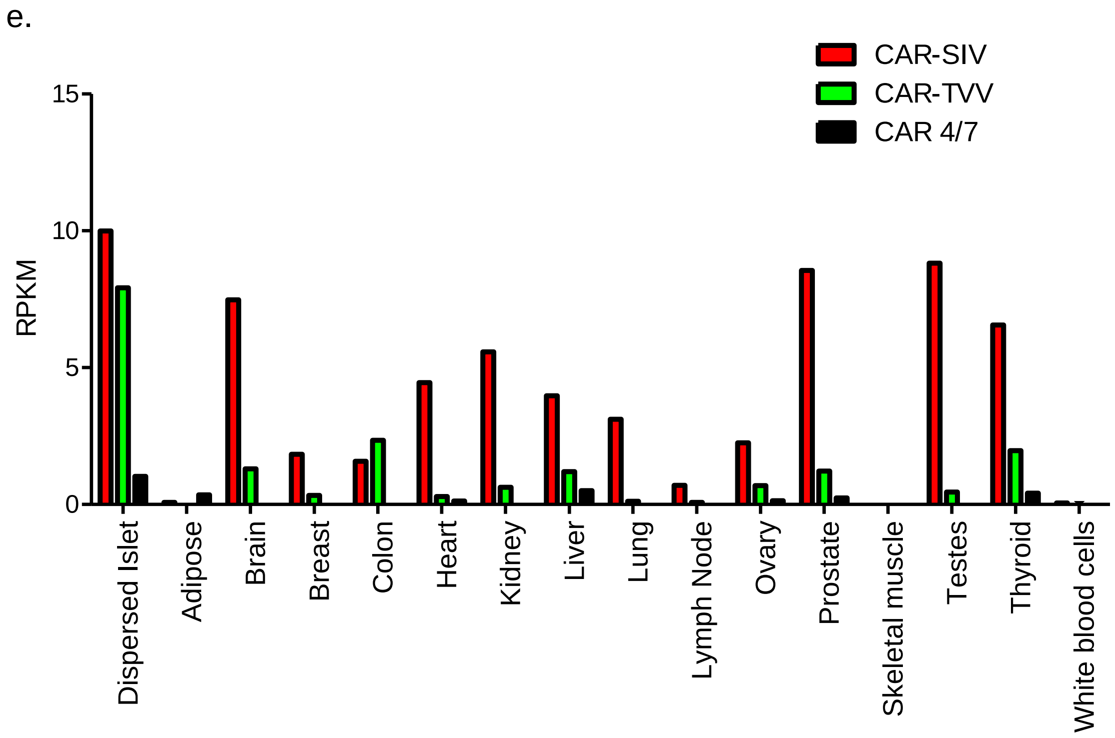

ESM Fig 4: CAR-SIV subcellular localisation and distribution in human tissue. Immunohistochemical staining demonstrating either surface membrane or granular distribution of CAR-SIV in different human tissues. **(a)** Testes, **(b)** Bladder small cell carcinoma, **(c)** Brain **(d)** Stomach. **(e)** RNA-seq analysis of *CXADR* isoforms supports the expression profile of CAR-SIV in dispersed human islets and various other normal human tissues. CAR-SIV is highly expressed in dispersed islets, brain, kidney, heart, prostate, testis and thyroid when compared to CAR-TVV and CAR 4/7.

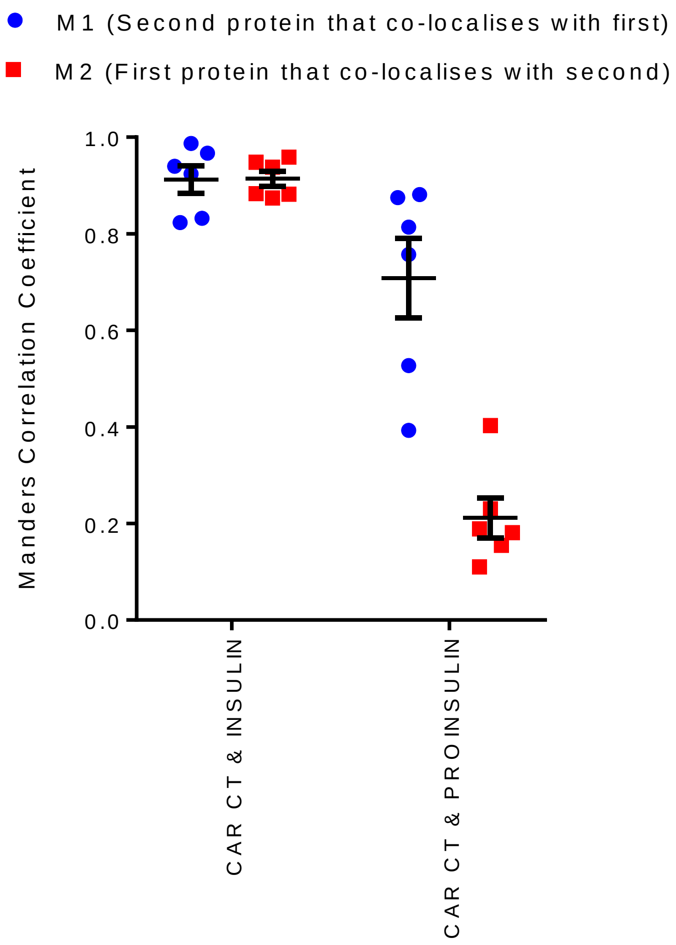

ESM Fig. 5 Manders Colocalisation Coefficient (MCC) analysis. This analysis allows us to assess how much of the proinsulin or insulin immunostaining is coincident with CAR-SIV and vice versa. This more detailed analysis has shown that the vast majority of CAR-SIV co-localises with insulin (MCC: 0.914±0.016) and that the same is also true for insulin co-localising with CAR-SIV (MCC: 0.912±0.028). This is consistent with them both being present in mature insulin secretory granules. In contrast, CAR-SIV does not co-localise to the same extent with proinsulin (MCC: 0.211±0.042), presumably because the majority of CAR-SIV is in the mature granules (which is supported by the immuno-EM quantitative analysis). In contrast, a higher proportion of proinsulin co-localises with CAR-SIV (MCC: 0.708±0.082) and this is likely to represent both CAR-SIV and pro-insulin in the immature granules as these emerge from the trans-Golgi.

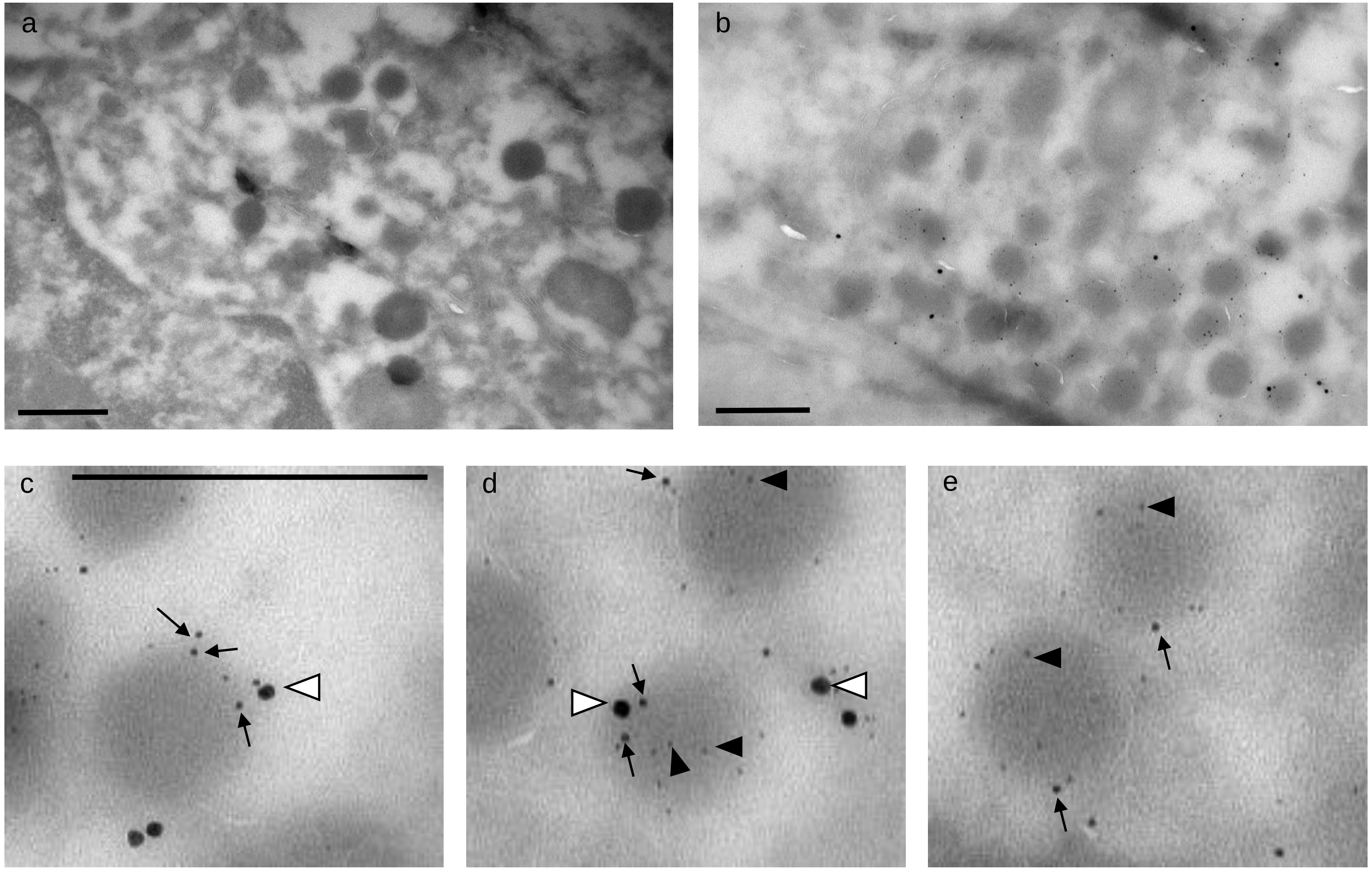

ESM Fig. 6 Cryo-immune EM analysis of insulin granules at different stages of maturation. Low magnification image (a) demonstrates no labelling of the pancreatic tissue when exposed to unconjugated gold particles and bridging antibodies. (b-e) Immunogold labelling of insulin (5nm gold; black triangle); CAR-SIV (10nm gold; black arrow) and proinsulin (20nm gold; white triangle) in thin frozen sections of human pancreas tissue. The higher magnification images reveal (c) immature granules positive for proinsulin and CAR-SIV, (d) maturing granules positive for proinsulin, insulin and CAR-SIV and (e) mature granules positive for insulin and CAR-SIV. Scale bar: 500 nm

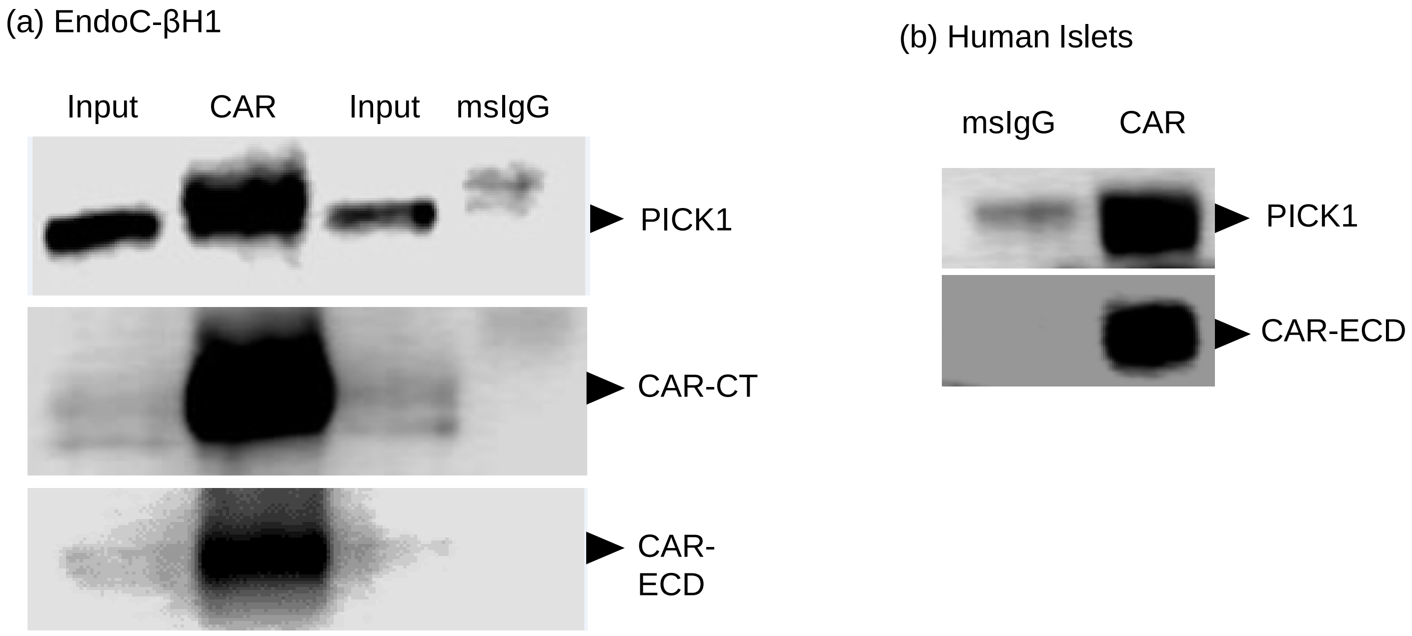

ESM Fig. 7 Immunoprecipitation of CAR from (a) EndoC-βH1 cells and (b) human islets also pulls down PICK1. Co-immunoprecipitation (see ESM Materials) was carried out with anti-CAR RmcB or anti-Mouse IgG (msIgG) as negative control. Immunoprecipitated proteins were analysed on Western blots probed with anti-PICK1. The membrane was then stripped and re-probed with anti-CAR CT or anti-CAR ECD. Input control (2%) was included as shown.
